# Functional brain connectivity related to surgical skill dexterity in physical and virtual simulation environments

**DOI:** 10.1117/1.NPh.8.1.015008

**Published:** 2021-03-03

**Authors:** Arun Nemani, Anil Kamat, Yuanyuan Gao, Meryem Yucel, Denise Gee, Clairice Cooper, Steven Schwaitzberg, Xavier Intes, Anirban Dutta, Suvranu De

**Affiliations:** a Rensselaer Polytechnic Institute, Center for Modeling, Simulation, and Imaging in Medicine, Troy, New York, United States; b Massachusetts General Hospital, Department of Surgery, Boston, Massachusetts, United States; c University at Buffalo School of Medicine and Biomedical Sciences, Buffalo, New York, United States

**Keywords:** functional connectivity, functional near-infrared spectroscopy, motor skills, fundamentals of laparoscopic surgery

## Abstract

**Significance:** Surgical simulators, both virtual and physical, are increasingly used as training tools for teaching and assessing surgical technical skills. However, the metrics used for assessment in these simulation environments are often subjective and inconsistent.

**Aim:** We propose functional activation metrics, derived from brain imaging measurements, to objectively assess the correspondence between brain activation with surgical motor skills for subjects with varying degrees of surgical skill.

**Approach:** Cortical activation based on changes in the oxygenated hemoglobin (HbO) of 36 subjects was measured using functional near-infrared spectroscopy at the prefrontal cortex (PFC), primary motor cortex, and supplementary motor area (SMA) due to their association with motor skill learning. Inter-regional functional connectivity metrics, namely, wavelet coherence (WCO) and wavelet phase coherence were derived from HbO changes to correlate brain activity to surgical motor skill levels objectively.

**Results:** One-way multivariate analysis of variance found a statistically significant difference in the inter-regional WCO metrics for physical simulator based on Wilk’s Λ for expert versus novice, F(10,1)=7495.5, p<0.01. Partial eta squared effect size for the inter-regional WCO metrics was found to be highest between the central prefrontal cortex (CPFC) and SMA, CPFC-SMA ($\eta^{2}=0.257$). Two-tailed Mann–Whitney U tests with a 95% confidence interval showed baseline equivalence and a statistically significant (p<0.001) difference in the CPFC-SMA WPCO metrics for the physical simulator training group (0.960±0.045) versus the untrained control group (0.735±0.177) following training for 10 consecutive days in addition to the pretest and posttest days.

**Conclusion:** We show that brain functional connectivity WCO metric corresponds to surgical motor skills in the laparoscopic physical simulators. Functional connectivity between the CPFC and the SMA is lower for subjects that exhibit expert surgical motor skills than untrained subjects in laparoscopic physical simulators.

## 1 Introduction

Surgical training has traditionally followed an apprenticeship-based model where technical skills are taught in the operating room.^1^^,^^2^ However, this approach is often costly, time-consuming, and presents significant adverse patient outcomes due to the trainee’s inexperience. Furthermore, with the advent of minimally invasive surgery and laparoscopic procedures, programs such as the Fundamentals of Laparoscopic Surgery (FLS) and the Fundamentals of Endoscopic Surgery (FES) have been adopted by the American Board of Surgery as accredited means for assessing technical surgical skills.^3^^–^^11^ Surgical skill assessment in these simulator-based training methods often utilizes rating scales, rudimentary performance metrics, or direct observation methods to rate and assess surgical task performance.^9^^,^^12^^–^^17^ While these metrics’ usage is standard of practice in surgical skill training and assessment, they have been cited for having low interpreter reliability and poor correlation of simulator-based performance metrics to clinical outcomes in the operating room.^2^^,^^18^^,^^19^

Compounding the lack of robust surgical skill assessment metrics, there is a lack of studies that comprehensively address the underlying neurophysiological responses to varying surgical motor skill levels. Current studies have shown the potential of non-invasive brain imaging to quantify cortical activation differences for subjects with varying degrees of surgical motor skills.^20^^–^^25^ These studies have shown significant differences in functional activation in the prefrontal cortex (PFC) (Refs. 20–32), and recently, in the primary motor cortex (M1) and the supplementary motor area (SMA)^33^ as well, due to their involvement in motor skill learning. However, the underlying temporal correlations between these anatomically separated cortical brain regions correlated to surgical motor skills have not yet been studied systematically. In this regard, functional connectivity methodologies can leverage such temporal correlations to classify or distinguish subjects.^34^ Indeed, techniques to quantify brain functional connectivity, such as wavelet coherence (WCO) and wavelet phase coherence (WPCO), have been utilized in multiple functional near-infrared spectroscopy (fNIRS) studies.^35^^–^^41^ WCO and WPCO analyses can objectively quantify functional connectivity and strong temporal correlations by determining significantly high common power and phase-locked behavior between two specific cortical channels;^36^^,^^42^ this approach can address the neurophysiological knowledge gap of surgical motor skill learning effects on the brain.

Herein, we report the inter-regional functional connectivity between the three above-mentioned cortical regions, namely, the PFC, the M1, and the SMA. Beyond the difference in activation levels of each of these cortical regions as demonstrated in Ref. 33, we hypothesize that the surgical motor skill levels will significantly affect the functional brain connectivity measured with WCO and WPCO during assessment using both virtual and physical surgical simulators. To test this hypothesis, subjects with varying degrees of surgical motor expertise performed a complex surgical training task on physical and virtual simulators while undergoing fNIRS imaging of oxygenated hemoglobin (HbO) changes for each brain region. To quantify inter-regional functional connectivity between cortical regions, WCO and WPCO were calculated from HbO time-series as they performed the surgical training task.

## 2 Methods and Materials

### 2.1 Subjects

Thirty-six right-handed subjects were recruited in this Institutional Review Board-approved study conducted at the Massachusetts General Hospital and the University at Buffalo. The subjects were split into two cohorts. The first cohort included novice and expert surgeons and the second cohort included training medical students. The second cohort was further divided into three distinct groups: FLS training group, virtual basic laparoscopic skills trainer (VBLaST) training group, and control group. An *a priori* power analysis, based on two-sample t-tests, was completed to determine the minimum number of samples required for both cohorts in this study. Using pilot study data and the power estimation software G*Power,^43^ we estimated conservative effect sizes for the FLS and VBLaST training groups for FLS and VBLAST task performance scores, d=5.67 and d=2.57, respectively.^25^ With a 95% confidence interval (CI) and a minimum power of 0.80, a minimum of eight subjects each for the expert and novice surgeon cohort group, four subjects for the FLS training group, three subjects for the VBLaST training group, and four subjects for the control group was estimated for this study.

All the participants were instructed on how to perform the task with standardized verbal instruction indicating the goal of the task and rules for the task completion. The optical probe was positioned on the participants with great care to avoid any hair between source/detector and scalp, and robust coupling with the skin. Each participant’s experimental protocol consisted of a block design of rest and stimulus period (surgical cutting task). Each surgeon performed five trials, whereas the control group performed three trials, as shown in the table below. The surgical cutting task was performed until the completion or stopped after 5 min. Then a rest period of one minute was given. This cycle of the rest period and task continued for the number of trials indicated above. Subject demographics are summarized in Table 1, and further details on subject recruitment, compensations, and other pertinent study replication details can be found in Nemani et al.^25^

**Table 1:** Study subject demographics and training procedures completed.

Cohort	# of subjects	Mean age	Training/certification	Average # of laparoscopic procedures	# of completed FLS pattern cutting trials	# of completed VBLaST pattern cutting trials
Expert surgeon	8	35	Postgraduate year 4 to 5 or attending surgeons	700	5	5
Novice surgeon	9	31	Postgraduate year 1 to 3	60	5	5
FLS training group	9	25	Medical school year 1 to 4	0	>100	0
VBLaST training group	8	24	Medical school year 1 to 4	0	0	>85
Control	5	26	Medical school year 1 to 4	0	3	3

### 2.2 Hardware and Study Design

We utilized two different surgical training simulators that employ the pattern cutting task. As a physical surgical trainer, we used the official FLS box trainer used in Board certification.^9^^,^^44^^,^^45^ As a representation of a virtual surgical trainer, we utilized the VBLaST, a virtual reality-based simulator that replicates the FLS training tasks.^25^^,^^46^^–^^50^ We employed a commercially available fNIRS system to measure functional brain activation during surgical training pattern cutting tasks (CW6 system, TechEn Inc., Massachusetts). Infrared light was delivered at 690 and 830 nm to eight different sources that were coupled to eight different short separation detectors and 16 long separation detectors. Each long separation detector was separated from its corresponding source by 30 to 40 mm to ensure depth specificity to the cortex. The short separation detectors were placed 8 mm away from each source to ensure that only superficial tissue, such as the scalp, skull, dura, and pial matter, were measured.

### 2.3 Protocol Design

All study participants were asked to perform the pattern cutting task. The objective was to use laparoscopic tools to cut a marked circle on a piece of gauze as accurately and quickly as possible. Each subject was instructed on how to perform the task using a standardized verbal dictation indicating the pattern cutting task’s rules and goals. Each session consisted of each subject performing the pattern cutting task with rest periods between each subsequent trial. Further information regarding study design can be found in Nemani et al.^25^

### 2.4 fNIRS Data Processing for the Hemodynamic Response Function

All fNIRS data processing were completed using HOMER2, a validated and published open-source software suite implemented in Matlab (Mathworks, Natick, Massachusetts), which provides a set of Matlab scripts used for analyzing fNIRS data.^51^ Prior to any data processing, data channels that exhibit low signal to noise ratios, namely, outside of the range of 80 to 140 dB, were excluded from the analysis. The modified Beer–Lambert law was used to convert the detectors’ raw optical data into optical density (hmrIntensity2OD). The fNIRS data can be contaminated with the inevitable motion artefacts due to the participant’s motion while doing the pattern cutting task. Any such large motion artefacts were corrected using principal component analysis (PCA) in HOMER2 (hmrMotionCorrectedPCA);^51^^–^^53^ however, no filters were applied to the time-series data to preserve the entire frequency bandwidth of each channel. PCA application assumes that any large motion has a dominant contribution to the variance of the fNIRS data, and because the first principal component will account for the largest proportion of that variance, which was removed from the original fNIRS data. Then, following the conversion of optical density to changes in oxy and deoxy-hemoglobin concentrations (hmrOD2Conc) with partial path-length factors of 6.4 (690 nm) and 5.8 (830 nm), the short separation channels (an inter-optode distance of 8 mm) were regressed from the long separation channels (an inter-optode distance of 30 to 40 mm) using a general linear model (GLM) in HOMER2 to remove systemic physiology originating from non-cortical superficial regions.^54^^,^^55^ Then, the hemodynamic response function (HRF) was estimated by the GLM approach in HOMER2 (hmrDeconvHRF_DriftSS) that uses ordinary least squares. The HRFs were calculated using a consecutive sequence of Gaussian functions as the temporal basis for the HRF.^54^^,^^56^^–^^58^ The result is a time-series that shows changes in HbO for each brain region that is specific to the cortical activity. The functional connectivity metrics were computed between each pair of HbO time-series from the following brain regions: left lateral prefrontal cortex (LPFC), central prefrontal cortex (CPFC), right lateral prefrontal cortex (RPFC), left medial primary motor cortex (LMM1), and SMA.

### 2.5 Wavelet Coherence and Wavelet Phase Coherence Metrics of Functional Connectivity

**Fig. 1:**
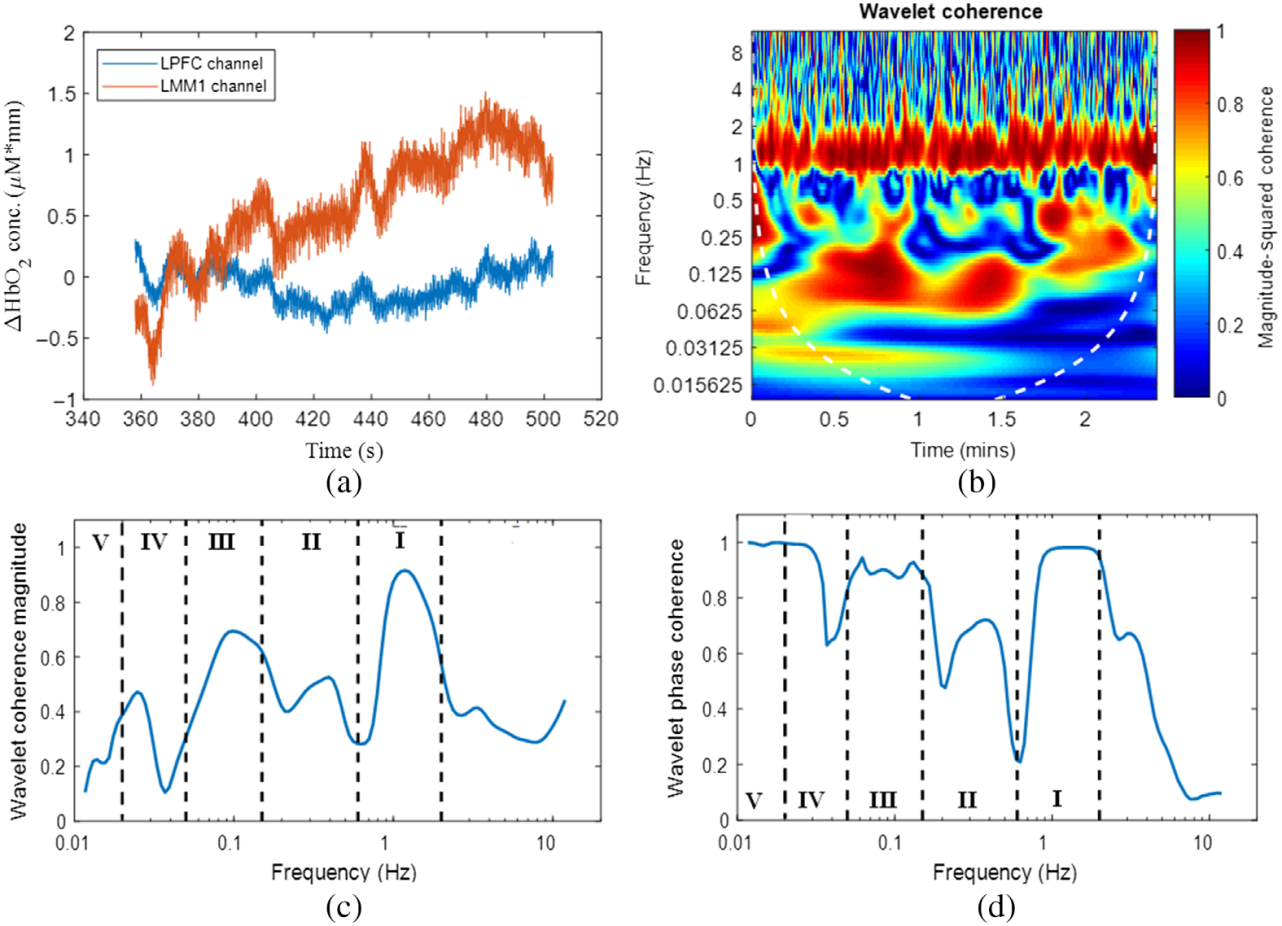
An illustrative example of WCO between two different fNIRS time-series data. (a) Timeseries data from the left lateral PFC (LPFC) and left medial M1 (LMM1) channels for a surgical expert during one FLS task trial. (b) WCO magnitude between the two time-series data in time and frequency domains. WCO magnitude values are shown via the color bar. Only values within the cone of influence range, indicated by a dashed white line, are included for WCO power magnitude and phase coherence calculations. (c) Time-averaged WCO magnitudes and (d) WPCO magnitudes between the two example time series shown in (a).

To objectively quantify functional connectivity between time series from different cortical regions, we utilize the WCO and WPCO metrics. WCO as a function of frequency is defined below:^59^^,^^60^

$$WCO(f)=\frac{\left[\frac{1}{N}\overset{N}{\underset{n=1}{\sum }}w_{1}(t_{n})w_{2}^{*}(t_{n})][\frac{1}{N}\overset{N}{\underset{m=1}{\sum }}w_{1}^{*}(t_{m})w_{2}(t_{m})\right]}{P_{1}(f)P_{2}(f)}$$ (1)

where $w_{1}$ and $w_{2}$ are complex oscillatory Morlet wavelet transforms of the first and second-time series, N is the total number of time steps of each time series, * is the complex conjugate, and $P_{1}(f)$ and $P_{2}(f)$ are the wavelet power at frequency f. The time-averaged WPCO is also defined below:^33^^,^^36^^,^^59^^,^^60^

$$WPCO(f)=\sqrt{{\left\langle \cos\;\Delta \phi (f)\right\rangle }^{2}+{\left\langle \sin\;\Delta \phi (f)\right\rangle }^{2}}$$ (2)

$$\left\langle \cos\;\Delta \phi (f)\rangle =\frac{1}{N}\overset{N}{\underset{n=1}{\sum }}\;\cos\;\Delta \phi (f,t_{n}\right)$$ (3)

$$\left\langle \sin\;\Delta \phi (f)\rangle =\frac{1}{N}\overset{N}{\underset{n=1}{\sum }}\;\sin\;\Delta \phi (f,t_{n}\right)$$ (4)

where $\Delta \varphi (f,t_{n})$ is the instantaneous phase difference between two complex oscillatory time series. The coefficients cos $\Delta \varphi (f,t_{n})$ and sin $\Delta \varphi (f,t_{n})$ is then time-averaged across the entire time series. The significance of these metrics is that they can objectively quantify correlations of two independent time series with specificity to the frequency and temporal changes.^35^ A value of 0 for both WCO and WPCO indicates that two time-series are entirely unrelated in phase changes and coherence magnitudes. A value of 1 for both WCO and WPCO indicates a significant linear relationship between the two time-series and that the oscillatory phase changes are significantly correlated.^35^^,^^38^^,^^61^^,^^62^ As shown in Table 2 below, the entire frequency bandwidth of the resulting WCO and WPCO vectors is split into five different intervals that are correlated to different physiological activities. Furthermore, results from WCO and WPCO analysis are shown for two examples of fNIRS time-series in Fig. 1. Figure 1(a) shows two example channels, the left lateral PFC and the left medial M1, for one subject while performing the FLS pattern cutting task. Figure 1(b) shows the corresponding WCO magnitude plot for each frequency and time step between the two example channels. Figures 1(c) and 1(d) are the time-averaged WCO and WPCO magnitudes. Furthermore, the frequency intervals are depicted to show the specific coherence magnitudes ranges for each associated physiology. It is worth noting that only WCO and WPCO values within the cone of influence, depicted as a shaded white line, are used for analysis due to edge effects that may bias the analysis. The inter-regional functional connectivity metrics (WCO and WPCO) were computed between LPFC and CPFC (LPFC-CPFC), between LPFC and RPFC (LPFC-RPFC), between LPFC and SMA (LPFC-SMA), between LPFC and LMM1 (LPFC-LMM1), between CPFC and RPFC (CPFC-RPFC), between CPFC and SMA (CPFC-SMA), between CPFC and LMM1 (CPFC-LMM1), between RPFC and SMA (RPFC-SMA), between RPFC and LMM1 (RPFC-LMM1), and between SMA and LMM1 (SMA-LMM1).

**Table 2:** Frequency bandwidth intervals with their associated physiology.^35^^–^^38^^,^^67^

Frequency interval	Frequency range (Hz)	Associated physiology
I	0.6 to 2	Cardiac activity
II	0.15 to 0.6	Respiratory activity
III	0.05 to 0.15	Myogenic smooth muscle activity
IV	0.02 to 0.05	Neurovascular coupling and autonomic control in the cortex
V	0.005 to 0.02	Nitric oxide-related endothelial metabolic activity

### 2.6 Statistical Testing

The inter-regional functional connectivity metrics (WCO and WPCO) from the first cohort of novice and expert surgeons were used to conduct a one-way multivariate analysis of variance (one-way MANOVA) in SPSS version 27 (IBM) to determine whether there is any significant difference in the inter-regional (i.e., LPFC-CPFC, LPFC-RPFC, LPFC-SMA, LPFC-LMM1, CPFC-RPFC, CPFC-SMA, CPFC-LMM1, RPFC-SMA, RPFC-LMM1, and SMA-LMM1) functional connectivity metrics between novice and expert surgeons using Wilks’ Lambda. The Shapiro-Wilk test was used to test normality for each of the dependent variables (i.e., inter-regional functional connectivity metrics) for the independent variable, novice, and expert. Also, the Levene test was used to test homogeneity of variance. All the significance levels were set at p<0.01. Then, to determine how the dependent variables (i.e., inter-regional functional connectivity) differ for the independent variable (novice versus expert), partial eta squared effect size was used and with alpha correction with Bonferroni correction. The dependent variable with the largest partial eta squared effect size was selected to investigate medical students’ surgical training effects. Nonparametric two-tailed Mann–Whitney U tests were utilized within a 95% CI to determine the baseline equivalence and a significant difference in WCO and WPCO metrics following surgical training between the training group and the control group.

## 3 Results

### 3.1 Functional Connectivity Differences between Expert and Novice Surgeons

**Fig. 2:**
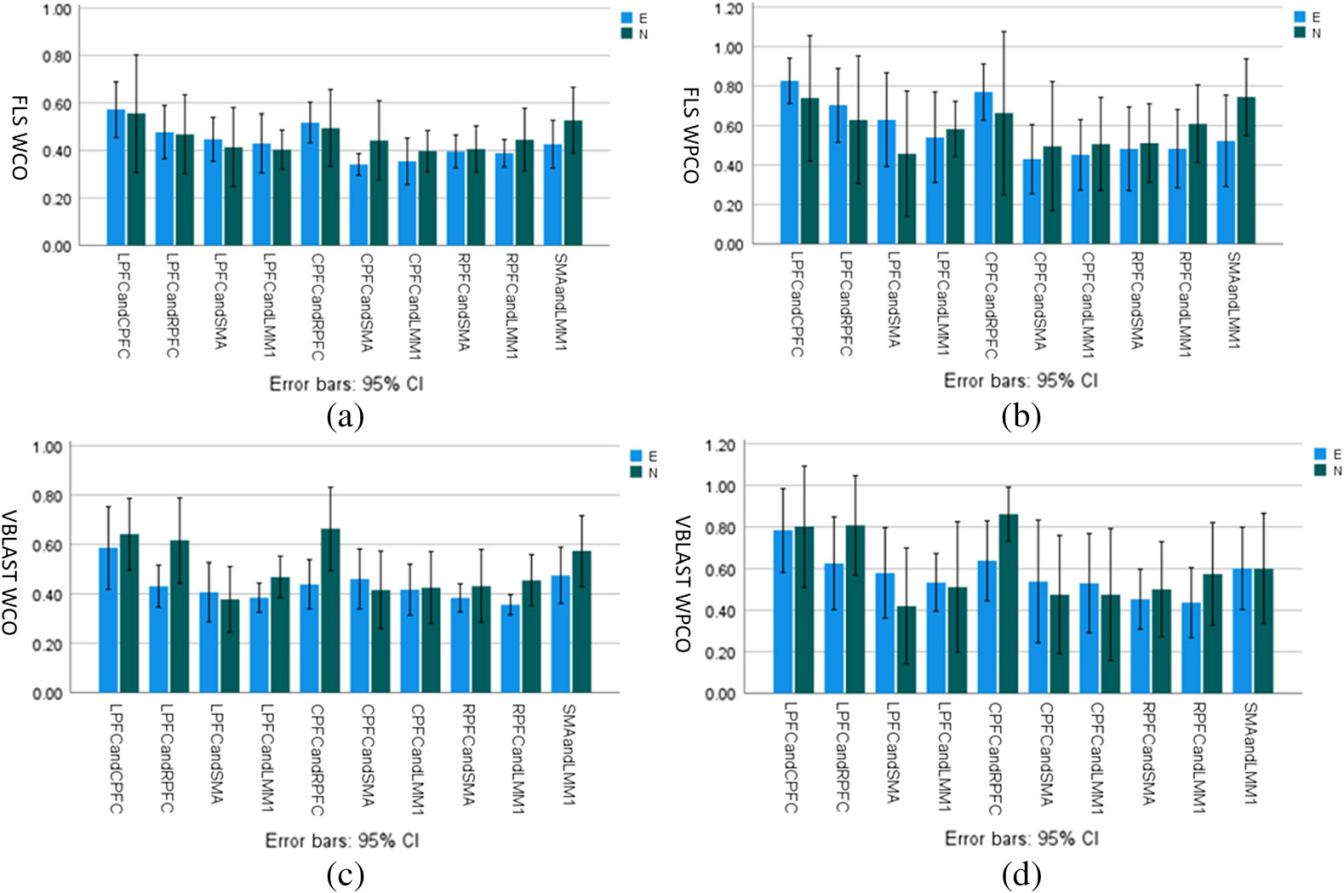
WCO and WPCO magnitude changes between expert (E) and novice (N) surgeons on physical (FLS) and virtual (VBLAST) simulators. (a)–(b) WCO magnitudes and WPCO magnitudes for FLS experts (blue) vs. novices (green) within the neurovascular coupling activity frequency range (0.02 to 0.05 Hz). (c)–(d) WCO magnitudes and WPCO magnitudes for VBLaST experts (blue) versus novices (green) within the neurovascular coupling activity frequency range. Error bars represent a 95% CI.

To investigate significant inter-regional functional connectivity differences between expert and novice surgeons on physical (FLS) or virtual (VBLaST) simulators, we report in Fig. 2 the mean WCO and WPCO metrics with error bars representing 95% CIs for LPFC-CPFC, LPFC-RPFC, LPFC-SMA, LPFC-LMM1, CPFC-RPFC, CPFC-SMA, CPFC-LMM1, RPFC-SMA, RPFC-LMM1, and SMA-LMM1. Shapiro–Wilk test showed that each of the inter-regional functional connectivity metrics is normally distributed at a significance level of p<0.01. Also, Levene’s test of equality of error variance was satisfied at a significance level of p<0.01. One-way MANOVA found statistically significant difference only in the inter-regional WCO metrics for physical (FLS) simulator based on Wilk’s Λ for expert versus novice, F(10,1)=7495.5, p<0.01. Partial eta squared effect size for the inter-regional WCO metrics was found to be highest between the CPFC and SMA, CPFC-SMA ($\eta^{2}=0.257$). The other Partial Eta Squared effect sizes were, LPFC-CPFC: $\eta^{2}=0.003$, LPFC-RPFC: $\eta^{2}=0.001$, LPFC-SMA: $\eta^{2}=0.024$, LPFC-LMM1: $\eta^{2}=0.015$, CPFC-RPFC: $\eta^{2}=0.013$, CPFC-LMM1: $\eta^{2}=0.058$, RPFC-SMA: $\eta^{2}=0.005$, RPFC-LMM1: $\eta^{2}=0.121$, SMA-LMM1: $\eta^{2}=0.195$. Figure 2 shows that the CPFC-SMA WCO and WPCO metrics were higher in novice than experts in physical (FLS) simulator while higher in expert than a novice in virtual (VBLAST) simulators. All MANOVA results are provided in Figures S1 to S4 in the Supplementary Materials.

### 3.2 CPFC-SMA Functional Connectivity Changes during FLS Surgical Training

**Fig. 3:**
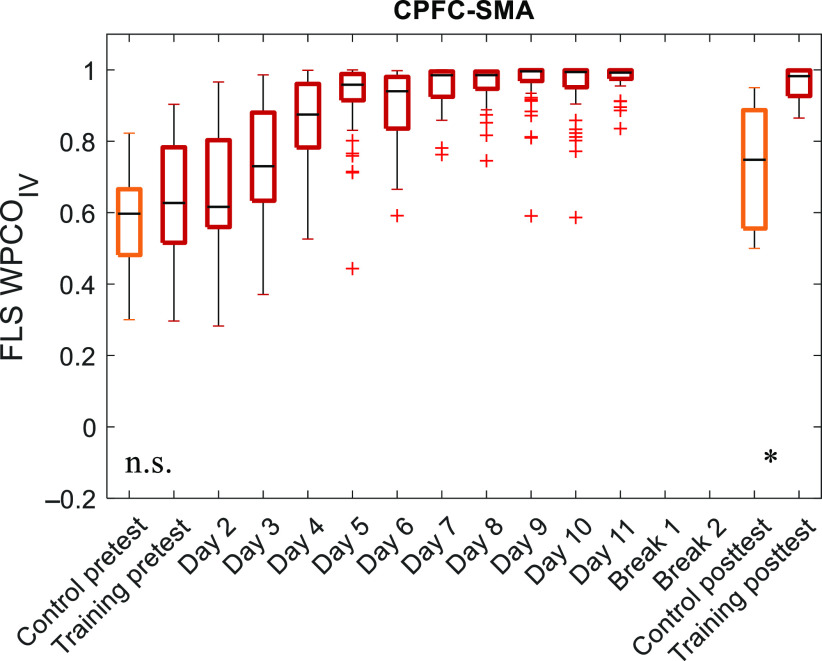
Longitudinal WPCO with FLS surgical skill training. WPCO magnitudes within the neurovascular coupling activity frequency range (0.02 to 0.05 Hz) between the CPFC and SMA channels for the untrained control group and FLS training group during surgical training over ten consecutive days.

Since functional connectivity changes between the CPFC and SMA, CPFC-SMA was responsive to increased surgical motor skill proficiency in expert versus novice during physical (FLS) simulator task, so we calculated CPFC-SMA WCO and WPCO metrics for FLS practice in medical student trainees. Figure 3 shows the longitudinal functional connectivity results of the FLS training group and the control group. Two-tailed Mann–Whitney U tests with a 95% CI showed baseline equivalence and a statistically significant (p<0.001) difference in the WPCO metric between the FLS training group (0.960±0.045) and the untrained control group (0.735±0.177).

## 4 Discussion

While surgical simulators are significantly gaining ground for surgical skill training and assessments,^2^ the underlying neurological mechanisms or functional connectivity between correlated cortical regions are mostly unstudied. This study compares the functional connectivity of cortical regions associated with fine motor skills for subjects with varying degrees of surgical motor skill. We found a statistically significant (p<0.01) difference in the inter-regional WCO metrics for physical (FLS) simulator task for expert versus novice surgeons based on Wilk’s Λ. The inter-regional WCO metric was found to have the highest Partial Eta Squared effect size for the CPFC and SMA. Furthermore, a statistically significant (p<0.001) difference in the CPFC-SMA WPCO metric was found for the physical (FLS) simulator training group when compared to the untrained control group following baseline equivalence and then training for 10 consecutive days (in addition to the pretest and posttest days).

Inter-regional functional connectivity within the neurovascular coupling frequency range (0.02 to 0.05 Hz) is postulated to be related to neuronal communication. Neurovascular coupling is the interaction between neural activity and vascular response in terms of regional cerebral blood supply and HbO during brain activity. The interaction between HbO time-series between two brain regions can be assessed using various methods, including WCO, a measure of the correlation between two time-series, and WPCO based on the degree of coincidence of instantaneous phase over the entire time-series.^63^ Lower WCO and WPCO metrics of functional connectivity between CPFC and SMA (Fig. 2) in an expert when compared to a novice are postulated to be related to more implicit knowledge-based physical (FLS) surgical task performance in experts.^64^ PFC has been shown to be engaged during explicit motor-sequence learning while implicit knowledge activates SMA,^64^ so CPFC-SMA functional connectivity indicates an interplay between explicit motor-sequence learning and implicit knowledge during FLS surgical task performance and learning in novice. Our study provided preliminary evidence on CPFC-SMA functional connectivity to assess this interplay between motor and frontal regions in experts versus novices in physical (FLS) simulators where tactile and proprioceptive feedbacks are available.^64^ However, inter-regional PFC WCO and WPCO metrics, including LPFC-RPFC and CPFC-RPFC, were found to be more relevant during virtual (VBLaST) surgical task performance (Fig. 2) that was higher in novices than experts. Here, higher inter-regional PFC WCO and WPCO metrics may indicate explicit motor-sequence learning^64^ in novices during VBLaST surgical task performance primarily using visual feedback.

During surgical tasks in physical and virtual simulators, our neuroimaging approach utilized the most recent advances in portable functional brain imaging using fNIRS with increased specificity to cortical tissue due to short separation regression.^57^^,^^58^ Such methods provide a more accurate estimation of the cortical tissue’s hemodynamics during complex bimanual surgical tasks in virtual and physical simulators (Refs. 55, 65, and 66), which have not been reported previously. Our results quantified inter-regional functional connectivity solely based on WCO and WPCO metrics that showed promise in assessing surgical motor skill proficiency and can be utilized for learning assessment during surgical training in the future. The differences in cortical activation and inter-regional functional connectivity between physical (FLS) or virtual (VBLaST) simulators need further investigation.

## 5 Conclusion

This study showed that functional connectivity changes based on WCO and WPCO metrics corresponded to the surgical motor skill proficiency, and these connectivity changes were in the neurovascular coupling frequency range in the cortical regions. Our study showed that surgical experts and surgically trained subjects exhibited functional activation correlations and the instantaneous phase’s coincidence over the CPFC and SMA time-series. These results further our understanding of neural correlates of the interplay between motor and frontal regions related to fine motor learning associated with surgical training and can be used for future assessment paradigms.
