# Functional brain connectivity related to surgical skill dexterity in physical and virtual simulation environments (Erratum)

**DOI:** 10.1117/1.NPh.8.3.039801

**Published:** 2021-07-27

**Authors:** Arun Nemani, Anil Kamat, Yuanyuan Gao, Meryem Yucel, Denise Gee, Clairice Cooper, Steven Schwaitzberg, Xavier Intes, Anirban Dutta, Suvranu De

**Affiliations:** a Rensselaer Polytechnic Institute, Center for Modeling, Simulation, and Imaging in Medicine, Troy, New York, United States; b Massachusetts General Hospital, Department of Surgery, Boston, Massachusetts, United States; c University at Buffalo School of Medicine and Biomedical Sciences, Buffalo, New York, United States

## Abstract

The erratum corrects the Acknowledgments section of the published article.

This article [*Neurophotonics* **8**(1), 015008 (2021) doi: 10.1117/1.NPh.8.1.015008] was originally published on 3 March 2021 with incomplete information in the Acknowledgments section.

Acknowledgments (original):

This work was supported by the National Institute of Biomedical Imaging and Bioengineering (NIBIB) under Grant Nos. 1R01EB014305, NHLBI 1R01HL119248, and NCI 1R01CA197491 grants. The authors would like to thank the attending surgeons, residents, and medical student subjects and their dedication to this study. We would also like to thank Arthur “Buzz” DiMartino and his team at TechEn for graciously providing us support with the CW6 spectrometer.

Acknowledgments (corrected):

This work is supported by the National Institutes of Health (NIH), Award Nos. NIBIB 1R01EB014305, NHLBI 1R01HL119248, and NCI 1R01CA197491; and the Medical Technology Enterprise Consortium (MTEC), Award No. W81XWH2090019 (2020-628). The authors would like to thank the attending surgeons, residents, and medical student subjects and their dedication to this study. We would also like to thank Arthur “Buzz” DiMartino and his team at TechEn for graciously providing us support with the CW6 spectrometer.

The information was corrected on 8 July 2021.

