# Supplementary material for: Functional brain connectivity related to surgical skill dexterity in physical and virtual simulation environments
Source: Neurophotonics. 2021 Mar 3;8(1):015008. doi: 10.1117/1.NPh.8.1.015008 (PMC7927423; doi:10.1117/1.NPh.8.1.015008)
Supplement: Supplementary file 1 [file NPh_008_015008_SD001.pdf]

Multivariate Tests[a]

Effect		Value	F	Hypothesis df	Error df	Sig.	Partial Eta Squared
Intercept	Pillai's Trace	1.000	4933164.40[b]	10.000	1.000	.000	1.000
	Wilks' Lambda	.000	4933164.40[b]	10.000	1.000	.000	1.000
	Hotelling's Trace	49331643.98	4933164.40[b]	10.000	1.000	.000	1.000
	Roy's Largest Root	49331643.98	4933164.40[b]	10.000	1.000	.000	1.000
EvsN	Pillai's Trace	1.000	7495.465[b]	10.000	1.000	.009	1.000
	Wilks' Lambda	.000	7495.465[b]	10.000	1.000	.009	1.000
	Hotelling's Trace	74954.645	7495.465[b]	10.000	1.000	.009	1.000
	Roy's Largest Root	74954.645	7495.465[b]	10.000	1.000	.009	1.000

a. Design: Intercept + EvsN

b. Exact statistic

Figure S1: One-way MANOVA in SPSS version 27 (IBM, USA) to determine whether there are any significant difference in the inter-regional functional connectivity WCO metrics between novice (N) and expert (E) surgeons during physical (FLS) simulator pattern cutting task.

Multivariate Tests[a]

Effect		Value	F	Hypothesis df	Error df	Sig.	Partial Eta Squared
Intercept	Pillai's Trace	.997	33.871[b]	10.000	1.000	.133	.997
	Wilks' Lambda	.003	33.871[b]	10.000	1.000	.133	.997
	Hotelling's Trace	338.713	33.871[b]	10.000	1.000	.133	.997
	Roy's Largest Root	338.713	33.871[b]	10.000	1.000	.133	.997
EvsN	Pillai's Trace	.996	27.586[b]	10.000	1.000	.147	.996
	Wilks' Lambda	.004	27.586[b]	10.000	1.000	.147	.996
	Hotelling's Trace	275.862	27.586[b]	10.000	1.000	.147	.996
	Roy's Largest Root	275.862	27.586[b]	10.000	1.000	.147	.996

a. Design: Intercept + EvsN

b. Exact statistic

Figure S2: One-way MANOVA in SPSS version 27 (IBM, USA) to determine whether there are any significant difference in the inter-regional functional connectivity WPCO metrics between novice (N) and expert (E) surgeons during physical (FLS) simulator pattern cutting task.

Multivariate Tests[a]

Effect		Value	F	Hypothesis df	Error df	Sig.	Partial Eta Squared
Intercept	Pillai's Trace	.992	25.849[b]	10.000	2.000	.038	.992
	Wilks' Lambda	.008	25.849[b]	10.000	2.000	.038	.992
	Hotelling's Trace	129.244	25.849[b]	10.000	2.000	.038	.992
	Roy's Largest Root	129.244	25.849[b]	10.000	2.000	.038	.992
EvsN	Pillai's Trace	.724	.524[b]	10.000	2.000	.801	.724
	Wilks' Lambda	.276	.524[b]	10.000	2.000	.801	.724
	Hotelling's Trace	2.619	.524[b]	10.000	2.000	.801	.724
	Roy's Largest Root	2.619	.524[b]	10.000	2.000	.801	.724

a. Design: Intercept + EvsN

b. Exact statistic

Figure S3: One-way MANOVA in SPSS version 27 (IBM, USA) to determine whether there are any significant difference in the inter-regional functional connectivity WCO metrics between novice (N) and expert (E) surgeons during virtual (VBLAST) simulator pattern cutting task.

Multivariate Tests[a]

Effect		Value	F	Hypothesis df	Error df	Sig.	Partial Eta Squared
Intercept	Pillai's Trace	.996	51.561[b]	10.000	2.000	.019	.996
	Wilks' Lambda	.004	51.561[b]	10.000	2.000	.019	.996
	Hotelling's Trace	257.804	51.561[b]	10.000	2.000	.019	.996
	Roy's Largest Root	257.804	51.561[b]	10.000	2.000	.019	.996
EvsN	Pillai's Trace	.972	6.944[b]	10.000	2.000	.132	.972
	Wilks' Lambda	.028	6.944[b]	10.000	2.000	.132	.972
	Hotelling's Trace	34.720	6.944[b]	10.000	2.000	.132	.972
	Roy's Largest Root	34.720	6.944[b]	10.000	2.000	.132	.972

a. Design: Intercept + EvsN

b. Exact statistic

Figure S4: One-way MANOVA in SPSS version 27 (IBM, USA) to determine whether there are any significant difference in the inter-regional functional connectivity WPCO metrics between novice (N) and expert (E) surgeons during virtual (VBLAST) simulator pattern cutting task.
